# Current Evidence for a Bidirectional Loop Between the Lysosome and Alpha-Synuclein Proteoforms

**DOI:** 10.3389/fcell.2020.598446

**Published:** 2020-11-17

**Authors:** Norelle C. Wildburger, Anna-Sophia Hartke, Alina Schidlitzki, Franziska Richter

**Affiliations:** ^1^Department of Pharmacology, Toxicology, and Pharmacy, University of Veterinary Medicine, Hanover, Germany; ^2^Center for Systems Neuroscience, Hanover, Germany

**Keywords:** alpha-synuclein, proteoforms, lysosome, Parkinson disease, GCase

## Abstract

Cumulative evidence collected in recent decades suggests that lysosomal dysfunction contributes to neurodegenerative diseases, especially if amyloid proteins are involved. Among these, alpha-synuclein (aSyn) that progressively accumulates and aggregates in Lewy bodies is undisputedly a main culprit in Parkinson disease (PD) pathogenesis. Lysosomal dysfunction is evident in brains of PD patients, and mutations in lysosomal enzymes are a major risk factor of PD. At first glance, the role of protein-degrading lysosomes in a disease with pathological protein accumulation seems obvious and should guide the development of straightforward and rational therapeutic targets. However, our review demonstrates that the story is more complicated for aSyn. The protein can possess diverse posttranslational modifications, aggregate formations, and truncations, all of which contribute to a growing known set of proteoforms. These interfere directly or indirectly with lysosome function, reducing their own degradation, and thereby accelerating the protein aggregation and disease process. Conversely, unbalanced lysosomal enzymatic processes can produce truncated aSyn proteoforms that may be more toxic and prone to aggregation. This highlights the possibility of enhancing lysosomal function as a treatment for PD, if it can be confirmed that this approach effectively reduces harmful aSyn proteoforms and does not produce novel, toxic proteoforms.

## Parkinson Disease

Parkinson disease (PD) is the second most common neurodegenerative disorder after Alzheimer disease (AD) and is the most common movement disorder (de Lau and Breteler, 2006). Clinically, patients present with four main motor symptoms—resting tremor, bradykinesia (slowness of voluntary movement), rigidity, and loss of postural reflexes—as first described by English physician James Parkinson (Parkinson, 1817; Fahn and Sulzer, 2004). These symptoms and related exclusionary criteria comprise the differential diagnosis of PD in the clinic today (Poewe et al., 2017).

Globally, PD currently affects 6.1 million individuals (Collaborators, 2018) or ∼2% of the population older than 65 (de Lau and Breteler, 2006; Poewe et al., 2017). PD before age 50 is rare (Twelves et al., 2003; Pringsheim et al., 2014; Poewe et al., 2017), but the incidence increases with age by ∼5–10-fold starting in the sixth decade of life (Twelves et al., 2003; Van Den Eeden et al., 2003; Savica et al., 2013; Pringsheim et al., 2014; Poewe et al., 2017) when individuals are still professionally engaged with many more active years. After diagnosis, individuals face a prolonged 7–15-year period of increasing disability (Golbe and Leyton, 2018). Age is the greatest risk factor for PD, so as the lifespan of the population continues to increase, so too will the frequency of PD (Dorsey et al., 2007). Beyond age, non-genetic aspects such as sex and environmental factors influence the lifetime risk of PD (de Lau and Breteler, 2006; Poewe et al., 2017), but the precise etiology of ∼90% of all PD cases is largely unknown.

It was nearly a century after Parkinson’s clinical description before Fritz Heinrich Lewy published findings of proteinaceous aggregations or “Lewy bodies” (LBs) in the PD brain (Lewy, 1912) later named after him (Trétiakoff, 1919). Braak and Braak subsequently used LBs to develop the widely accepted staging system of PD progression (Braak et al., 2003). The staging system is based on the stereotypical spread of LBs throughout brain and is currently used in research and clinics to provide a definitive diagnosis of PD (Dickson et al., 2009). Cellular and molecular PD investigations began in 1960 when it was discovered that PD brains exhibited dramatic reduction in dopamine (DA) levels (Ehringer and Hornykiewicz, 1960). The motor symptoms that typify PD manifest after a ∼50–60% loss of substantia nigra dopaminergic neurons (Kirik et al., 2002). As a result, the DA precursor L-3,4-dioxyphenylalanine (L-DOPA) became the first therapeutic agent aimed at compensating for the loss of endogenous DA (Birkmayer and Hornykiewicz, 1962).

The current standard of care is still DA replacement therapy, but this does not always improve motor and/or non-motor symptoms and cannot halt PD progression. The utility of L-DOPA and similar therapeutics is limited since all exhibit a “wearing-off” phenomenon and are associated with motor and non-motor side effects (e.g., hallucinations) (Cacabelos, 2017). Furthermore, it is widely acknowledged that by the time motor symptoms appear, there is little that can be done to halt or reverse disease pathogenesis. This is reinforced by pathological studies by Braak and colleagues who showed that LB brain lesions were evident long before the first motor symptoms can be clinically detected (Braak et al., 2003). However, the first significant breakthrough in several decades came in 1997 Spillanti and colleagues showed that LBs were strongly reactive to alpha-synuclein (aSyn) antibodies (Spillantini et al., 1997, 1998). This connected the pathophysiological finding of LBs in PD patients to the small presynaptic protein aSyn. Contemporaneous genetic studies reinforced that hypothesis by linking a missense point mutation in aSyn to familial PD (Polymeropoulos et al., 1997). As a result, PD came to be characterized as a neurodegenerative disease with abnormal accumulation of insoluble aSyn—a synucleinopathy (McCann et al., 2014).

## aSYN: Small Protein, Enigmatic Function, and Many Diseases

Synuclein was discovered as a 143 amino-acid-long, neuron-specific presynaptic protein in *Torpedo californica* (an electric ray) (Maroteaux et al., 1988) and was found to have a highly homologous cDNA counterpart in rat brain (Maroteaux et al., 1988). Studies in AD brain revealed the human homolog of this protein, which was termed “non-Aβ component of AD amyloid precursor” (NACP) (Ueda et al., 1993). The group also purified a second, nearly identical but smaller protein called NAC from AD brain (Ueda et al., 1993). NACP later became known as aSyn, and the smaller protein NAC was termed beta-synuclein (bSyn) (Jakes et al., 1994). The last member of the synuclein family is BCSG1, now known as gamma-synuclein (gSyn) (Ji et al., 1997; Lavedan et al., 1998).

Despite its well-known role in synucleinopathies such as PD, dementia with LBs, and multiple system atrophy, the physiological function of aSyn remains enigmatic. The high concentrations of aSyn at presynaptic terminals suggest a role in synaptic release and plasticity, but genetic ablation of both aSyn and bSyn (Chandra et al., 2004) or all three synucleins (Anwar et al., 2011) in mice did not have profound effects in synaptic ultrastructure, plasticity, or phenotype. Electrochemical experiments in the triple knock-out model showed that DA release was specifically elevated in the dorsal striatum (Anwar et al., 2011), while DA levels were decreased in the double knock-out model (Abeliovich et al., 2000; Chandra et al., 2004). While its precise function is unknown, the current evidence suggests that synucleins are necessary for neurotransmitter release—particularly DA—and synaptic vesicle recycling.

Since aSyn is the main component in LBs (Spillantini et al., 1997, 1998) that neuropathologically characterize PD and other synucleinopathies, aberrant accumulation rather than ablation of the protein is likely what leads to a pathological phenotype. The genetic link between the aSyn missense A53T mutation and a heritable form of PD (Polymeropoulos et al., 1997) strengthened the hypothesis that aSyn is a key player in PD. Since then, research efforts by many groups identified five additional point mutants (E46K, A30P, H50Q, G51D, and A53E) linked to familial PD (Eriksen et al., 2005; Reed et al., 2019), followed by the discovery of a triplication mutation in the aSyn gene (*SNCA*) (Singleton et al., 2003), and then *SNCA* duplications (Chartier-Harlin et al., 2004). These discoveries drew attention to the gene dosage of *SNCA* and its effects on age of onset and disease severity. These point mutations in aSyn ultimately result in aberrant protein accumulation, but likely through different mechanisms such as faster aggregation kinetics and perturbations in the cellular machinery responsible for protein turnover (Eriksen et al., 2005; Xilouri et al., 2016). However, *SNCA* mutations are rare. Most cases of PD are idiopathic (onset > 60 years of age) but largely present with the same clinical and neuro-pathological features as familial PD (onset < 50 years of age).

Since PD is a progressive disease and the greatest risk factor is age, the combination of gradual modifications in aSyn and age-related deterioration of cellular degradation machinery may underlie the late-onset pathology of sporadic PD (Xilouri et al., 2016). Modified aSyn may impair protein degradation, and deficits in protein degradation machinery due to aging may result in accumulation of aSyn that can undergo modification or aggregation into toxic species that inhibit their own degradation or that of other substrates (Cuervo et al., 2004; Xilouri et al., 2016). In this review, we will examine what is currently known about the complex interplay between aSyn, its many proteoforms (Smith et al., 2013), and lysosomal protein degradation.

## Cellular Degradation in the Lysosome

**FIGURE 1:**
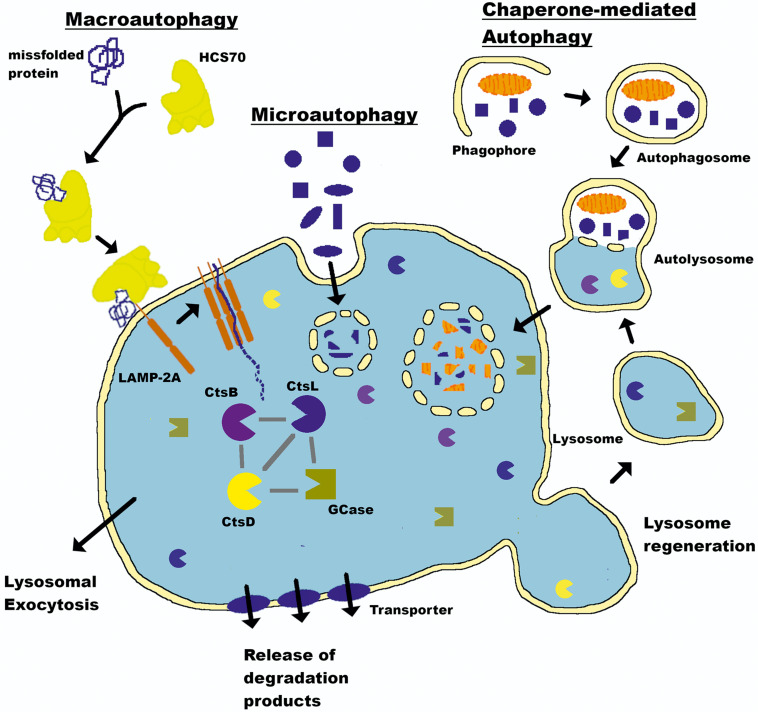
Autophagy-lysosomal pathway (ALP). The lysosome is a key cellular organelle for the degradation of proteins maintaining a balance between synthesis and clearance. Three pathways—macroautophagy, chaperone-mediated autophagy (CMA), and microautophagy—converge on this central hub to deliver cargo for degradation. The lysosome is a central mechanism by which the production of aSyn in the brain is controlled (see text for details). LAMP-2A, Lysosome-associated membrane glycoprotein 2; HSC70, heat shock cognate 70; CtsL, Cathepsin L; CtsD, Cathepsin D; CtsB, Cathepsin B; GCase, Glucocerebrosidase.

The proteasome system typically degrades proteins with short half-lives (Etlinger and Goldberg, 1977; Bigelow et al., 1981; Glickman and Ciechanover, 2002), while proteins with half-lives > 10 h are degraded by the autophagy-lysosomal pathway (ALP) (De Duve et al., 1955; Dice, 2000; Klionsky and Emr, 2000). The ALP comprises three distinct pathways: macroautophagy, which we will refer to as autophagy throughout this review; chaperone-mediated autophagy (CMA); and microautophagy. All three deliver intracellular and internalized extracellular constituents to the lysosome for degradation (Figure 1). Lysosomes are acidic (pH 4.6) cytoplasmic organelles containing hydrolytic enzymes that degrade intracellular components and are responsible for maintaining an appropriate balance between protein synthesis and degradation (De Duve et al., 1955).

The lysosome directly mediates microautophagy through membrane invaginations and uptake of proximal cytoplasmic material (Muller et al., 2000). This mechanism is seemingly random and poorly understood. There is no evidence that aSyn is degraded by microautophagy, and this component will not be discussed further (Xilouri et al., 2016). Conversely, autophagy and CMA are complex multistep processes requiring extensive intracellular signaling and synchronization to deliver their cargo to the lysosome in an ATP-dependent manner (Schellens et al., 1988; Arias and Cuervo, 2011; Kaushik and Cuervo, 2012). aSyn degradation occurs through both mechanisms (Webb et al., 2003; Cuervo et al., 2004; Lee et al., 2004). Autophagy begins with engulfment of cytoplasmic material in a double membrane structure called an autophagosome that ultimately fuses with the lysosome to deliver its contents. CMA does not require double membrane vesicle formation, but instead chaperone-mediated protein unfolding and trafficking to the lysosome via binding to a KFERQ-like CMA targeting motif. aSyn contains a pentapeptide sequence (_9__5_VKKDQ_99_) that is consistent with a CMA recognition motif (Dice, 1990; Cuervo et al., 2004). A review by Finkbeiner presents a detailed account of the ALP and its mechanisms (Finkbeiner, 2020).

## Lysosomal Linkages to PD

ALP impairment is increasingly recognized as a key pathological event in neurodegenerative disorders. Genetic studies strengthen the connection between PD and ALP as the majority of PD-associated genes are linked to lysosomal protein trafficking or lysosomal function (Chang et al., 2017; Klein and Mazzulli, 2018). Among these risk-enhancing genes, mutations in *GBA1*, the gene for the lysosomal hydrolase acid β-glucosidase (GCase), are the most common known genetic risk factor for PD (Sidransky et al., 2009; Bultron et al., 2010). Mutations in this gene are the main cause of a rare autosomal-recessive genetic disorder known as Gaucher disease (GD). The first association between GCase and PD was made when clinicians noted that patients with GD and carrier relatives developed parkinsonism (Goker-Alpan et al., 2004). A 2009 international multicenter study led by Ellen Sidransky established the high frequency of GBA1 mutations in PD (Sidransky et al., 2009). Today, the estimated prevalence of GBA1 mutations in PD patients ranges from 5 to 25%, and their presence increases the risk for PD by up to 20 times, depending on ethnicity (Lesage et al., 2010; Beavan and Schapira, 2013; Zhao et al., 2016) despite a low penetrance (Rosenbloom et al., 2011).

Mechanistically, loss of GCase function leads to accumulation of its substrates and metabolites, and these directly interfere with aSyn by promoting its toxic structural conversion to oligomers and amyloid fibrils (Zunke et al., 2018). However, these studies generally model total loss of GCase activity (i.e., Gaucher disease), but most GCase mutations linked to PD are heterozygotes. This reduces the total accumulation of substrates resulting from mutations in *GBA1*. Some mutations, like the intriguing case of E326K do not cause substrate accumulation or GD despite a high prevalence in PD patients (Nichols et al., 2009; Pankratz et al., 2012).

Concurrent with ALP dysfunction, aSyn accumulates and forms toxic species that further interfere with lysosomal function and neuronal health. It is conceivable that even in absence of GBA1 mutations, aSyn accumulation and pathology interferes with lysosomal function to create a pathogenic loop (Cookson, 2011; Dawson and Dawson, 2011; Mazzulli et al., 2011; Yap et al., 2013). Recent evidence suggests that alteration in GCase protein levels or enzyme activity is not a prerequisite for PD. Pharmacological inhibition of GCase exacerbates pathology in various primary cell lines and animal models, but alone cannot cause aSyn aggregation (Henderson et al., 2020). Misfolded and aggregated aSyn in the form of pre-formed fibrils (PFFs)—even at low levels—was sufficient to cause PD-like pathology. The results suggest that aSyn aggregation is a first step independent of GCase (Henderson et al., 2020), but that GCase modulates the vulnerability of cells to low levels of recombinant or native PFFs. GCase enzyme activity declined in the presence of PFFs. In a similar study, PFFs reduced GCase activity *in vitro* (Gegg et al., 2020).

Importantly, studies in postmortem brain samples support a role of lysosome dysfunction in PD. Key lysosomal enzymes such as Cathepsin D (CtsD) and lysosomal-associated membrane protein (LAMP)-2A are reduced in nigral neurons of subjects with PD, especially those containing aSyn inclusions (Chu et al., 2009). LBs are strongly immunoreactive for autophagosome markers, and lysosomal breakdown and autophagosome accumulation are evident in PD brain samples (Dehay et al., 2010). More specifically, GCase activity and protein levels are reduced in postmortem tissue. The most pronounced reduction is in the substantia nigra, and in *GBA1* mutation carriers (GCase activity loss of 58% in *GBA1* mutation carriers vs. 33% in sporadic PD) (Gegg et al., 2012). Interestingly, GCase is present in aSyn inclusions in other synucleinopathies such as Lewy body dementia (Goker-Alpan et al., 2011).

Further supporting the linkage between PD and lysosomal dysfunction is the association of deleterious lysosomal storage disorder (LSD) gene variants (excluding *GBA1*). Whole exome sequencing revealed that of the 54 genes examined, 56% of all PD cases at least one LSD causative gene variant alleles (e.g., *SMPD1, SLC17A5, ASAH1*, and *CTSD*) and 21% had multiple (Robak et al., 2017). Interestingly, several studies already implicate *CTSD* in PD for aSyn degradation (see section “aSyn Proteoforms: From Partial Proteolytic Degradation to Effects on Lysosomal Function” for aSyn proteoforms in the lysosome). The discovery sample set contained younger-onset PD patients (mean age∼41 years); however, the authors replicated the results in two independent cohorts that had a mean onset of ∼62 years, which represents the adult population and the age-associated risk for PD (Robak et al., 2017). A more recent study examining 23 LSD genes in a population with a mean onset age∼60 years confirm the significance of *GBA1* (Hopfner et al., 2020). The study found *CTSD* to be significant only prior to Bonferroni correction and did not replicate the *SMPD1* finding from previous reports (Robak et al., 2017; Alcalay et al., 2019).

The *ATP13A2* gene while significantly associated with PD risk (Hopfner et al., 2020) did not meet the significance criteria in the earlier study (Robak et al., 2017). Interestingly, another enzyme, *ATP10B*, involved in lysosomal membrane transport was recently reported as a PD risk via exome sequencing (Martin et al., 2020). *ATP10B* and *ATP13A2* mutations result in loss-of-function (Ramirez et al., 2006). The role of ATP13A2 is unclear, but ATP10B appears to be involved in lipid export from the lysosome like GCase. Such loss-of-function mutations would result in an accumulation of glucosylceramide in the lysosome similar to *GBA1* mutations that lead to GD. Supporting this, ablation of ATP10B in mouse cortical neurons elevated caspase-3 and following exposure to either 50 nM rotenone or 500 mM MnCl_2_, neuronal cell death increased (Martin et al., 2020). Finally, LSD genes with membrane function or localization, not examined by Robak and colleagues—*LAMP1, TMEM175*, and *VPS13C*—had a significant association with PD (Hopfner et al., 2020). Specifically, variants of *LAMP1* and *TMEM175* were predicted to be the most damaging in the context of PD (Hopfner et al., 2020).

Lysosomal dysfunction may be the link connecting specific neuronal susceptibility, amyloidosis, and aging in PD. As postmitotic cells, neurons heavily depend on efficient lysosomal degradation to prevent build-up of unfavorable protein species. Lack of efficient autophagy results in massive neuronal loss and formation of inclusion bodies that increase in size and number with aging (Komatsu et al., 2006). This complex interaction between aSyn and lysosomes likely originates from the complexity of this protein, which has highly diverse variants, as discussed below.

## Proteoforms

It was highly anticipated that the Human Genome Project would identify ∼100,000 genes, but the final number was closer to ∼20,000. Unexpectedly, the complexity of biological processes including aging and age-related diseases is driven by variations in protein levels. Proteins are the workhorse molecules of the cell, and the term proteoform encapsulates their numerous variations. Proteoform describes all protein variants of a single gene including posttranslational modifications (PTMs) and sequence variants (Smith et al., 2013; Aebersold et al., 2018; Smith and Kelleher, 2018). In the context of this review and for the field of neurodegeneration, we also include aggregates or multimers of aSyn and other aggregate-prone proteins within the scope of the term.

Modifications change proteins’ physicochemical and biochemical properties to produce a wide array of biomolecules that increase the range of functional outcomes in a biological system (Lichti et al., 2014; Aebersold et al., 2018; Smith and Kelleher, 2018). These proteoforms are either stable or dynamic and can locally or globally change in response to developmental signals, normal stimuli, aging, or disease—and in many cases the modifications drive these processes. One important example is the prion protein (PrP^*C*^) responsible for Creutzfeldt–Jakob disease in humans (Prusiner, 1982; Prusiner et al., 1993). PrP^*C*^ is present in normal healthy humans, but by mechanisms that are still not clear, it undergoes a structural transformation into the neurodegenerative PrP^*Sc*^ form that is capable of propagating and converting PrP^*C*^ to PrP^*Sc*^ (Bessen et al., 1995; Zabel and Reid, 2015).

Several studies have described the heterogeneity of amyloid-beta proteoforms in AD plaques (Mori et al., 1992; Roher et al., 1993a,b; Saido et al., 1995; Kuo et al., 1997; Portelius et al., 2010) and more recently oligomers or soluble high-molecular-weight aggregates (Brody et al., 2017; Wildburger et al., 2017). However, beyond influencing aggregation kinetics, the implications of these proteoforms remain to be elucidated. Likewise, research has identified several proteoforms of N-terminal-acetylated aSyn in both neuronal and non-neuronal cell lines (Bartels et al., 2011; Theillet et al., 2016) and human brain (Sarafian et al., 2013; Kellie et al., 2014). Phosphorylation of serine 87 and 129 have been reported in animals and human, respectively (Anderson et al., 2006; Oueslati et al., 2012). Phosphorylation of serine 129 (pS129) is a hallmark of LBs and Lewy neurites (Fujiwara et al., 2002; Saito et al., 2003) and thought to promote aSyn aggregation (Walker et al., 2013). However, another group suggested that pS129 inhibits aggregation (Paleologou et al., 2008) and pS129 is present in normal control brains, albeit at lower levels (Anderson et al., 2006; Kellie et al., 2014). Other PTMs include DA adducts (Conway et al., 2001), nitration (Giasson et al., 2000; Takahashi et al., 2002; Yamin et al., 2003; Chavarría and Souza, 2013), oxidation (Chavarría and Souza, 2013), deamidation (Kellie et al., 2014), and ubiquitination (Anderson et al., 2006). N- and C-terminal truncations have also been described (Li et al., 2005; Kellie et al., 2014), and loss of the hydrophilic C-terminal domain present at higher levels than full-length aSyn in LBs and synaptosomes (Li et al., 2005; Sarafian et al., 2013), though truncations may be part of normal aSyn metabolism (Anderson et al., 2006).

## aSyn Proteoforms and Their Impact on Lysosomal Function

aSyn is degraded by both the ALP and proteasome pathways (Webb et al., 2003), and the preferred mechanism may be dependent on cellular burden or accumulation as well as specific proteoforms (e.g., mutations, folding state, and PTMs) (Dehay et al., 2013; Moors et al., 2016). In this section, we will focus on the effects of aSyn proteoforms on lysosomal integrity and function.

### Phosphorylation

The addition of a phosphoryl group (PO_3_^–^) to biomolecules is the one of the most well-studied PTMs. Phosphorylation can occur on serine (S), threonine (T), and tyrosine (Y) side chains and is traditionally viewed as a molecular on/off switch, but it can also have consequences for protein-protein binding characteristics and/or subcellular localization (Lichti et al., 2014). aSyn has a number of available phosphorylation sites (18 in total; 4 S, 4 T, and 10 Y), but only a few have been described and investigated. S129 phosphorylation (pS129) is thought to be the most important, as early immunohistologic studies of PD brains estimated that ∼90% of aSyn in LBs is phosphorylated at this residue in humans (Fujiwara et al., 2002; Saito et al., 2003; Anderson et al., 2006) and animals models (Neumann et al., 2002; Takahashi et al., 2003). In the normal brain, pS129 is present, but to a much lesser extent (Anderson et al., 2006; Muntane et al., 2012; Kellie et al., 2014), suggesting a close association between this PTM and aSyn aggregation (Smith et al., 2005).

Martinez-Vicente et al. investigated the effects of pS129 phosphorylation using an *in vitro* model of CMA-mediated transport (Martinez-Vicente et al., 2008). A S129E phosphomimetic aSyn bound to—but was unable to cross—the membranes of isolated lysosomes (Martinez-Vicente et al., 2008). *In vitro* phosphorylated aSyn recapitulated these results although with more variability owing to the presence of phosphatases. Neither the presence of phosphomimetic nor *in vitro* phosphorylated aSyn at the lysosomal membrane impeded the binding and degradation of other CMA substrates such as glyceraldehyde 3-phosphate dehydrogenase (GAPDH), indicating a functional lysosome (Martinez-Vicente et al., 2008). Interestingly, lysosomal matrix components could degrade S129E aSyn upon exposure, suggesting that the lysosomal enzymes are capable of degrading modified aSyn, yet they were unable to do so because of a lack of membrane translocation. Clue to this translocation failure come from experiments using a 2 M excess of GAPDH, a well-characterized CMA substrate (Aniento et al., 1993). GAPDH displaced lysosomal bound S129E aSyn, suggesting that CMA failure for this proteoform is due to low binding affinity to CMA receptors at the lysosomal membrane (Martinez-Vicente et al., 2008). Based on these results, it would appear that this aSyn proteoform prevents its own degradation (Martinez-Vicente et al., 2008). Inhibition of autophagy with chloroquine increased pS129 aSyn levels by 2-fold in SH-SY5Y human neuroblastoma cells, suggesting that autophagy plays a role in elevated aSyn as a substrate for phosphorylation, albeit to a lesser extent then CMA (Machiya et al., 2010). It is reasonable to conclude that deficits in these pathways would lead to elevated pS129 aSyn levels and ultimately aggregation and deposition into LBs.

If CMA or autophagy does not degrade pS129 or S129E, one would expect its phosphosilent counterpart, S129A, to be effectively processed by these pathways. Yet Tenreiro et al. (2014) found that phosphosilent S129A aSyn failed to activate autophagy in budding yeast. As a result, there was a significant increase in Triton X-100 insoluble aggregates and oligomers (Tenreiro et al., 2014). In line with this result, evidence suggests that pS129 may promote or activate autophagy through Polo-like kinase 2 (PLK2)-mediated phosphorylation of monomeric aSyn (Oueslati et al., 2013). PLK2 binds to aSyn in an ATP-dependent manner in HEK cells and promotes the clearance of aSyn and pS129 aSyn via autophagy (Oueslati et al., 2013).

Protein kinases are a superfamily of ∼500 proteins (3% of the human genome) (Manning et al., 2002). Screening all known kinases for similar effects on aSyn is impractical, but studies have examined G protein-coupled receptor kinases (GRKs). Previous reports describe aSyn as a GRK substrate (Pronin et al., 2000; Sakamoto et al., 2009). Expression of GRK family members did not recapitulate the effect of PLK2 on aSyn, suggesting that S129 phosphorylation and subsequent autophagy of aSyn is a specific feature of PLK2. In a genetic PD rat model, PLK2 adeno-associated virus (AAV) overexpression of PLK2 reduced aSyn accumulation by 55.8% compared to kinase dead PLK2, resulting in reduced dopaminergic neurodegeneration and associated motor symptoms (Oueslati et al., 2013). However, the S129A phosphosilent proteoform of human aSyn accumulated in rat brain neurons even with PLK2 overexpression, failing to activate autophagy, which is similar to the results in yeast described by Oueslati et al. (2013) and Tenreiro et al. (2014). Based on these findings, we can conclude that phosphorylation of S129 aSyn plays a key role in autophagy, but whether phosphorylation activates or inhibits autophagy remains unclear.

Finally, other aSyn phosphorylation sites may also affect its turnover by lysosomal degradation. Using site-directed mutagenesis to individually silence all four Y residues (Y→A), Choi and colleagues determined that phosphorylation of Y136 (a CtsD cleavage site) (Hossain et al., 2001; Choi et al., 2012) was most effective at promoting binding to Hsc70, a critical mediator of CMA, compared to unphosphorylated GST-aSyn and the other three aSyn Y residues (Choi et al., 2012).

### Oxidation

Oxidation is a covalent modification that can modify numerous amino acids reversibly (oxidation or sulfoxide) or irreversibly (sulfone). Oxidation modifies proteins either directly by reactive oxygen species or indirectly by oxidative stress reactions, leading to numerous structural and functional consequences. Protein oxidation is associated with age, and accumulation of oxidized proteins is involved in numerous diseases (Berlett and Stadtman, 1997; Li et al., 2004). Martinez-Vicente et al. used isolated rat liver lysosomes to directly test the effects of oxidized aSyn on CMA binding, uptake, and degradation (Martinez-Vicente et al., 2008). Binding of oxidized aSyn to lysosomal membranes was not significantly different from its wild-type (WT) or phosphomimic (S129E) counterparts (Martinez-Vicente et al., 2008). Oxidized aSyn induced only a modest decrease in CMA uptake without affecting CMA function. GAPDH could displace oxidized aSyn, but oxidized aSyn was able to successfully compete with GAPDH for CMA degradation (identified by percent inhibition of GAPDH degradation) at levels similar to that of WT aSyn (Cuervo et al., 2004; Martinez-Vicente et al., 2008). It is important to note that due to the lack of mass spectrometry protein sequencing, the amino acid residues oxidized after 90 min (longer periods resulted in aggregation) *in vitro* remain undetermined.

### DA Modification

DA is a key neurotransmitter involved in several distinct dopaminergic pathways in the brain. In the context of PD, DA is the primary neurotransmitter responsible for motor modulation in the basal ganglia. Loss of dopaminergic neurons in the substantia nigra pars compacta (SNpc), a component of the basal ganglia, results in the clinical features of PD. DA is normally contained within the synaptic vesicles of neurons and upon stimulation is released into the cytosol where it binds to postsynaptic receptors. Cytosolic DA can readily oxidize to dopaminochrome, even without the aid of metal-ion catalysis (Linert et al., 1996; Munoz et al., 2012). Consequently, DA release and re-uptake are tightly controlled.

aSyn exposure to DA or dopaminochrome (oxidized DA and the precursor to neuromelanin) (Conway et al., 2001; Norris et al., 2005) increased protein binding to the lysosomal membrane compared to unmodified aSyn (Martinez-Vicente et al., 2008). The translocation of DA- and dopaminochrome-modified aSyn were similarly impaired to the phosphorylated and phosphomimic proteoforms, but unlike the phosphorylated and oxidized aSyn proteoforms, the binding affinities of both DA-modified aSyns were sufficient to prevent displacement by a 2 M excess of GAPDH. It also inhibited the CMA of GAPDH, suggesting that it inhibited its own degradation and that of other proteins (Martinez-Vicente et al., 2008). In contrast, phosphorylated and oxidized aSyn could inhibit their own degradation but not that of other substrates (Martinez-Vicente et al., 2008). This behavior of DA- and dopaminochrome-modified aSyn is strikingly similar to that of mutated A53T and A30P aSyn implicated in familial PD (Cuervo et al., 2004; Martinez-Vicente et al., 2008) in that they show: (1) tight binding to the lysosomal membrane, (2) lack of translocation into lysosomes, and (3) CMA inhibition of other biological substrates (Martinez-Vicente et al., 2008) (see section “Mutations”). These effects were specific to DA-modified aSyn, as mutation of DA-sensitive residues Y_12__5_EMPS_129_ to F_12__5_AAFA_129_, respectively (Norris et al., 2005), did not affect lysosomal binding or uptake (Martinez-Vicente et al., 2008).

Given the drastic change in aSyn induced by DA or dopaminochrome, the authors sought to verify these effects in primary ventral midbrain (VM) neurons. They used L-DOPA to increase cytosolic DA to levels required for adduct formation on aSyn as previously determined from their *in vitro* data. CMA was inhibited by nearly 50% in a cell culture population that was only 40% dopaminergic (Martinez-Vicente et al., 2008). The effect was dependent on endogenous aSyn expression, as CMA in VM cultures from aSyn-/- mice was unimpaired. L-DOPA (increases DA levels) (Martinez-Vicente et al., 2008) and the tyrosine hydroxylase (TH) inhibitor α-methyl-p-tyrosine (αMT, decreases DA levels) (Xu et al., 2002) had no effect on primary cortical neurons and completely inhibited aSyn-induced apoptosis in dopaminergic neurons, respectively. Blocking CMA with RNA interference (RNAi) against LAMP-2A decreased cell viability, but no more than the addition of L-DOPA (Martinez-Vicente et al., 2008). Finally, DA-modified aSyn accumulated in the lysosomes of retinoic acid (RA)-differentiated SH-SY5Y cells but not DA-insensitive aSyn mutants (Martinez-Vicente et al., 2008). These results suggest that high concentrations of endogenous DA were required in addition to aSyn (Xu et al., 2002; Martinez-Vicente et al., 2008). However, the nature of the species that inhibits CMA remains unclear (Martinez-Vicente et al., 2008).

Using the familial mutant A53T, Conway and colleagues screened a commercially available compound library for inhibitors of aSyn fibrilization, and some were validated against WT aSyn (Conway et al., 2001). Intriguingly, DA and L-DOPA inhibited monomeric WT aSyn fibrillation (i.e., stabilized aSyn oligomers called protofibrils in that study), while the effect was reversed by antioxidants (e.g., Na_2_S_2_O_5_, *N*-acetyl cysteine, and deferoxamine) (Conway et al., 2001; Norris et al., 2005). The level of DA-adducted aSyn was determined to be ∼10% of total aSyn by both mass spectrometry and gel filtration. Yet as little as 1–3% DA-aSyn was sufficient to promote aSyn oligomer stabilization (Conway et al., 2001). DA also inhibited fibril formation of aSyn A53T at an equimolar ratio (Norris et al., 2005).

Evidence of this DA-mediated effect on aSyn was lacking until Mazzulli and colleagues used TH mutants in WT and A53T aSyn-expressing RA-differentiated SH-SY5Y cells. TH mutants lack the catecholamine feedback inhibition binding site (_3__7_RR_38_→GG or _3__7_RR_38_→EE), which results in elevated cytosolic L-DOPA, DA, and 3,4-dihydroxyphenylacetic acid (DOPAC, a DA metabolite) without increasing total TH protein levels (Mazzulli et al., 2006). The numbers of thioflavin S aggregates visualized by immunofluorescence decreased in both WT and A53T aSyn-expressing cells. In the latter cells, the amount of Triton X-100-insoluble aggregates declined significantly. Using size-exclusion chromatography and western blotting, the authors confirmed that the decrease in A53T aSyn fibrillary aggregates was due to an increase in higher molecular weight aSyn oligomers in TH mutants. This effect occurred at the cellular level because the addition of DA during cell lysis did not promote aSyn oligomer formation. Inhibition of TH and thus DA synthesis with either αMT (0.5 mM) or NSD 1015 also abrogated oligomer formation, suggesting the effects were DA-dependent. Interestingly, in the study by Mazzulli et al. (2006), the increase in aSyn oligomers was innocuous to cell viability. In contrast, DA-modified aSyn was toxic, but with αMT (1 mM), survival of RA-differentiated SH-SY5Y expressing WT aSyn improved with lysosomal function (Xilouri et al., 2009). Xilouri et al. did not examine the effects of αMT on A53T aSyn, but it would seem that DA-modified WT aSyn, perhaps stabilized in an oligomeric form as suggested by others (Conway et al., 2001; Norris et al., 2005; Mazzulli et al., 2006), is responsible for reduced CMA function and increased toxicity.

Since aSyn lacks cysteine and tryptophan residues, Conway et al. speculated that DA adducted non-covalently to aSyn via tyrosine-derived radical coupling (Conway et al., 2001). Site-directed mutations of the four tyrosines, one histidine, and three of the four methionines (methione-1 is needed for bacterial protein expression) of aSyn demonstrated that DA stabilization of oligomers is independent of single or multiple residues (Norris et al., 2005). Yet, mutation of all five amino acid residues, Y_12__5_EMPS_129_ that contain one tyrosine and one methionine at the C-terminus was sufficient to counteract DA’s inhibitory effects (Norris et al., 2005). Further investigations by Norris et al. revealed that the oxidation product of DA, dopaminochrome, was just as effective at inhibiting fibrilization (Norris et al., 2005). These aSyn modifications are reversible under strongly denaturing conditions *in vitro*, though how this would be possible *in vivo* is unclear. The observations of DA- and dopaminochrome adduct-mediated stabilization of aSyn oligomers provide a possible mechanistic link between CMA impairment and DA-aSyn proteoforms. The brain contains high levels of aSyn (∼1% of total brain protein), and the SNpc has high DA levels. These results offer a possible explanation for the particular vulnerability of the SNpc to aSyn toxicity and the relative sparing of the ventral tegmental area in PD (Conway et al., 2001; Xu et al., 2002; Martinez-Vicente et al., 2008). As noted previously, the killer may well be in the house (Chesselet, 2003).

### Aggregates

While aSyn has been considered an intrinsically disordered monomer *in vivo* (Weinreb et al., 1996; Davidson et al., 1998; Fauvet et al., 2012; Theillet et al., 2016) recent reports have challenged this notion. They describe aSyn as a stable, spherical homo-tetramer (∼55 kDa) 3–3.5 nanometers in diameter (Bartels et al., 2011; Dettmer et al., 2013). Despite uncertainty about its native conformation, the general consensus is that aSyn aggregates, whether they be dimers, trimers, or higher-order aggregates (e.g., oligomers and fibrils), are the pathological species in PD (Winner et al., 2011). How these various assemblies influence and disrupt lysosomal function is only beginning to be uncovered after recent discoveries linking lysosomal genes to PD (Plotegher and Duchen, 2017; Klein and Mazzulli, 2018).

Like their monomer and dimer counterparts, both nitrated and non-nitrated aSyn oligomers bind to the lysosomal membrane but do not translocate into the lysosome (Martinez-Vicente et al., 2008). A 2 M excess of GAPDH could displace non-nitrated but not nitrated aSyn aggregates from the lysosomal membrane (Martinez-Vicente et al., 2008). This suggests nitrated and aggregated aSyn bind with high affinity to lysosomal membranes and prevent their own degradation. Even with degradation inhibited, aggregated aSyn does not reduce lysosomal CMA activity (Martinez-Vicente et al., 2008).

In contrast, COS-7 cells can effectively clear aSyn aggregates induced by the mitochondrial inhibitor rotenone. After COS-7 cells expressing aSyn are exposed to rotenone for ≥ 72 h, the clearance of these aggregates diminishes substantially (Lee et al., 2004). Using a centrifugation process, Lee and colleagues determined that fibrils, but not oligomers, are resistant to clearance. The mechanism of aSyn oligomer clearance in COS-7 cells appears to be mediated by lysosomes due to dose-dependent inhibition of oligomer removal upon treatment with bafilomycin (Baf) (Lee et al., 2004), which inhibits autophagy by disrupting the lysosomal pH gradient (Bowman et al., 1988). Supporting this data, E64, an irreversible cysteine-protease inhibitor that affects lysosomal proteases, also dose-dependently inhibits aSyn oligomer degradation. Imaging the lysosome with immunofluorescence and electron microscopy, the authors identified aSyn aggregates inside the lysosome, suggesting that they are able to translocate across the membrane unlike the aggregates described by Martinez-Vicente et al. (2008). The oligomers described in this work range from ∼50 to 200 kDa on western blot analysis, whereas the size range of oligomers described by Martinez-Vicente and colleagues is unclear.

While these studies indicate that the lysosome is still intact, others have reported that a heterogeneous combination of *in vitro*-produced aSyn aggregates ruptures intracellular vesicles (Freeman et al., 2013). This was evident by the relocalization of galectin 3, a sugar-binding protein present on the interior membrane of vesicles (Ray et al., 2010), in both human SH-SY5Y neuroblastoma cells and rat dopaminergic neuronal N27 cell lines expressing mCherry-Galectin3 (chGal3). Since aSyn aggregated *in vitro*, monomers were not likely responsible for the measured effect. This was confirmed by the addition of freshly resuspended aSyn that failed to induce any vesicle rupture (Freeman et al., 2013). To identify which type of intracellular vesicle ruptured, the authors used immunofluorescence microscopy and found that the lysosomal marker LAMP-2 colocalized with galectin 3 after aSyn aggregate treatment of SH-SY5Y cells (Freeman et al., 2013). aSyn typically but not always associated with the lysed vesicle. In N27 cells, this association was at the periphery of the ruptured vesicle.

To investigate the species responsible for vesicle rupture, Flavin et al. (2017) produced *in vitro* aggregates consisting of oligomers and fibrils and several other structural forms using recombinant WT and mutant aSyn (A30P, E46K, G51D, and A53T). Treating SH-SY5YchGal3 cells with fibrils of these aSyn proteoforms showed that they all induced vesicle rupture to the same extent. Not unexpectedly, aSyn oligomers were unable to rupture SH-SY5Y vesicle membranes (Flavin et al., 2017) as they can bind to the lysosomal membrane (Martinez-Vicente et al., 2008) and are internalized (Lee et al., 2004). Yet, in human induced pluripotent stem cell (hiPSC)-derived dopaminergic neurons, PFFs (commonly used to describe aSyn oligomers) were capable of inducing vesicle lysis (Flavin et al., 2017). aSyn fibrils were not tested in hiPSC cells. It is important to note that what the authors refer to as oligomers and PFFs appear to describe two distinct structure conformations of aSyn, even though PFFs usually refer to aSyn oligomers.

In human neuroglioma H4 cells and differentiated human mesencephalic cells, the propensity of extracellularly added aSyn to accumulate intracellularly along different lysosomal routes increased with greater size (fibrils > oligomers > monomers). All aggregated forms of aSyn colocalized with lysosomal markers (LAMP-1 and LAMP-2A), but oligomers and fibrils had a lower degree of association with LysotrackerRed and p62 (Hoffmann et al., 2019). Decreased colocalization with the Lysotracker indicates a low degree of association with the lysosomal compartment. Levels of p62 reflect lysosome degradation efficiency after autophagosome fusion (Klionsky et al., 2008). Morphologically, aSyn oligomers and fibrils caused lysosome enlargement similar to observations by Flavin et al. (2017). To confirm functional impairment, activity levels of the lysosomal enzyme CtsD decreased significantly in oligomer- and fibril-treated H4 cells (Hoffmann et al., 2019). These data indicate reduced aSyn degradation by autophagy (Hoffmann et al., 2019).

RA-differentiated SH-SY5Y cells resemble postmitotic neuron-like cells and do not require rotenone for aSyn aggregation like COS-7 cells. Aggregate formation increased when aSyn expression was induced at later days *in vitro*, linking cellular age with the propensity to form aggregates (Lee et al., 2004). In a similar vein, using a HEK293 aSyn-overexpressing line transduced with PFFs, Tanik et al. (2013) found that aSyn aggregates inhibit autophagy. In contrast to the work of Lee et al. (2004) but in agreement with Hoffmann et al. (2019), aSyn aggregates colocalized with the early markers of autophagy, p62 and LC3. However, aSyn aggregates did not co-localize with the lysosomal marker LAMP-1 (Tanik et al., 2013) as they did in H4 cells (Hoffmann et al., 2019), an effect recapitulated in primary neurons. This would suggest that the cells initiated the early steps of autophagy to clear aSyn aggregates, but these aggregates never arrived at the lysosome. Treatment with inhibitors and promotors of autophagy (3-MA and rapamycin, respectively), did not change intracellular aggregate levels (Tanik et al., 2013). Levels of intracellular aSyn remained constant after PFF treatment in a doxycycline-inducible promoter experiment compared to phosphate-buffered saline-treated cells. More importantly, the ratio of Triton X-100 soluble to insoluble aSyn decreased (Tanik et al., 2013). This effect was absent in primary neurons from aSyn knockout mice, demonstrating the result is dependent on the aggregation of endogenous aSyn and not exclusively exogenous PFF addition. Notably, the lysosome remained functional with a normal pH and the ability to degrade substrates (Tanik et al., 2013). In the context of this study, aggregates did not inhibit autophagy at the level of the lysosome but rather affected the earlier stages of autophagosome maturation (Tanik et al., 2013).

There are many documented aSyn proteoforms (Oueslati et al., 2010), but the impacts of these proteoforms on the ALP are still being elucidated. While the data conflict in some cases, likely due to the use of different cell lines, a pattern of overall ALP dysfunction emerges. It would appear that PTM-modified aSyn is resistant to degradation via CMA and in some instances autophagy. In other cases, PTMs stabilize aSyn oligomers (e.g., DA). Oligomers and other aggregates appear to disrupt autophagy and CMA to minor extents. However, the stage when this takes place and whether or not they enter or rupture the lysosome is still unclear. Precisely defining the mechanism of ALP disruption via aSyn proteoforms will require further investigation accounting for cellular and genetic background, mechanism of aSyn overexpression, and most importantly, unifying or standardizing the studied aSyn assemblies.

## Mutations

Currently there are six known *SNCA* missense mutations (A30P, E46K, A53T, H50Q, G51D, and A53E) that directly implicate aSyn as a causative agent in PD (Eriksen et al., 2005; Reed et al., 2019). Duplications and triplications of the *SNCA* gene also occur (Eriksen et al., 2005; Reed et al., 2019), but their effects on early onset PD are largely attributed to an increased “gene dosage” of aSyn. This rationale parallels that of APP gene triplication in Trisomy 21, which is considered to be the reason why nearly all individuals with Down syndrome develop neuropathology consistent with AD (Burger and Vogel, 1973; Wisniewski et al., 1985; Lai and Williams, 1989). In contrast, point mutations alter the primary structure of aSyn rather than increasing the genetic dose. Though rare, these single point mutations have profound consequences, particularly on the lysosome. Here we will review what is known about the direct or indirect effects of aSyn missense mutations on the ALP and potential mechanisms.

### A53T

The discovery of the first missense mutation in aSyn—A53T (Polymeropoulos et al., 1997)—led to the first animal models of PD and is the most well-studied PD-linked mutation. A53T exhibits a longer half-life than WT aSyn in RA-differentiated SH-SY5Y cells (74 h vs. ∼50 h), PC12 cells (∼60 h vs. ∼30 h) (Cuervo et al., 2004), and aged transgenic mice (Li et al., 2004). Critically, the increased measured half-life was not due to differential aggregation of A53T over WT aSyn, suggesting that the missense mutation either directly stabilizes the protein posttranslationally or that its functional (i.e., degradation) characteristics are altered.

In support of the latter hypothesis, A53T aSyn binds to intact lysosomes with a higher affinity than WT aSyn but is poorly internalized (Cuervo et al., 2004). Similar to studies in PC12 cells, A53T aSyn failed to interact directly with lysosomal enzyme CtsD, suggesting that A53T is *not* present *within* the lysosome (Stefanis et al., 2001). The binding of A53T aSyn occurred even at lower temperatures, which typically blocks binding and uptake of nearly all CMA substrates (Cuervo et al., 2004). Increased LAMP-2 co-immunoprecipitation with A53T aSyn compared to WT indicates high binding affinity of mutant aSyn to lysosomal membranes. A53T aSyn inhibited GAPDH degradation without impairing lysosomal function; lysosomal enzymes from disrupted lysosomes were still active and could degrade GAPDH (Cuervo et al., 2004). This suggests that like DA-aSyn, A53T aSyn blocks its own degradation and that of other CMA substrates (Cuervo et al., 2004) by occupying lysosomal binding sites with high affinity, preventing translocation of other substrates for degradation. In PC12 cells, mutant aSyn impaired CMA protein degradation as in isolated lysosomes (Cuervo et al., 2004). However, other data in PC12 cells showed that A53T reduced lysosome acidification (Stefanis et al., 2001). The presence of a high level of internalized substrate indicates cellular debris accumulation in non-functional lysosomes (Stefanis et al., 2001; Webb et al., 2003).

Xilouri et al. (2009), used undifferentiated PC12 cells expressing either A53T aSyn or mutant aSyn lacking the CMA-binding motif (DDQ/A53T aSyn) to examine the mechanisms of lysosomal dysfunction. The A53T mutant reduced total lysosomal degradation by 30%, whereas the DDQ/A53T mutant was similar to WT aSyn and control bgal-expressing cell (Xilouri et al., 2009). Further confirming that this reduction in protein degradation was due to CMA and not autophagy, the authors treated cells with 3-MA and found no difference between A53T and DDQ/A53T aSyn degradation (Xilouri et al., 2009). In SH-SY5Y cells, A53T reduced lysosomal degradation by ∼40%, but mutant aSyn lacking the CMA motif was similar to WT aSyn and control (Xilouri et al., 2009). In differentiated, postmitotic SH-SY5Y cells, A53T aSyn inhibited lysosomal degradation more markedly than in cycling PC12 and SH-SY5Y cells. Since both A53T and DDQ/A53T aSyn decreased lysosomal degradation, the impairment extended beyond CMA and affected autophagy (Xilouri et al., 2009).

Autophagy impairment resulted in elevated LC3-II levels, although this was limited to A53T not DDQ/A53T. The effect was similar in primary cortical cultures. Elevated levels of LC3-II suggest autophagosome accumulation (Xilouri et al., 2009). This would indicate a lack of autophagosome fusion with the lysosome or increased autophagy-mediated degradation as a cellular mechanism to remove mutant aSyn. However, in both differentiated SH-SY5Y cells and primary cortical neurons, the authors ascribe increased cell death to increased autophagy due to higher LC3-II levels (Xilouri et al., 2009).

### E46K

E46K was one of the *SNCA* missense mutations identified shortly after the discovery of A53T, further linking aSyn to familial PD (Zarranz et al., 2004). However, less is known about the role of this mutant in PD pathogenesis and ALP dysfunction. Evidence that the E46K aSyn mutant inhibits autophagy comes from studies of PC12 and HEK293 cells expressing the mutant protein (Yan et al., 2014). The authors found that total p62 levels, a reliable reporter for assessing autophagy activity, increased ∼24% compared to green fluorescent protein-expressing controls, and insoluble p62 levels increased nearly 200% (Yan et al., 2014). Accumulation of insoluble p62 is the most significant indicator of impaired autophagy. On confocal microscopy, p62 accumulation was clearly observed as discrete puncta throughout the cell cytoplasm. p62 exhibited reduced protein turnover after cycloheximide treatment (Yan et al., 2014, 2018). Most significantly, treatment with the autophagy inhibitor 3-MA equally increased p62 levels in control and E46K cells, demonstrating that the effect of this mutant was autophagy dependent (Yan et al., 2014). The lysosomes themselves were functional even with E46K aSyn colocalizing with Lysotracker (Yan et al., 2014). The E46K examined in the PC12 and HEK293 cells was presumably monomeric (Yan et al., 2014) since aggregated E46K in differentiated SH-SY5Y and N27 cells ruptured lysosomes after endocytosis (Freeman et al., 2013).

Autophagy is a multistep process, and deficiencies induced by monomeric E46K might occur further upstream compared to aggregated aSyn. Autophagosomes were significantly decrease in both E46K-expressing PC12 and HEK293 cells due to decreased synthesis rather than increased lysosomal degradation. The decline in LC3-II suggests that E46K inhibition of autophagy is due to reduced autophagosome formation (Yan et al., 2014). Interestingly, subsequent work by the same group demonstrated that E46K aSyn was present inside the lysosomes of PC12 cells as it colocalized with Lysotracker (Yan et al., 2018), raising the question of how mutant aSyn can enter the lysosome when autophagosomes are inhibited (Yan et al., 2014, 2018). One might speculate that E46K enters the lysosome via CMA as ALP activity increased (Yan et al., 2014), and perhaps this proteoform internally (rather than externally, like DA-modified aSyn) inhibits the degradation of other cellular substrates (Martinez-Vicente et al., 2008; Decressac et al., 2013). Studies in a *Drosophila* model of PD using pan-neuronal expression of E46K showed that this mutant is resistant to degradation (Sakai et al., 2019). E46K accumulated in both total protein and Triton X-100-soluble fractions (Sakai et al., 2019). While it was unclear where aSyn E46K accumulated in the work by Sakai and colleagues, it is possible that it occurred in the lysosome (Yan et al., 2018) reducing its own degradation and that of other substrates (Martinez-Vicente et al., 2008; Yan et al., 2014).

Mechanistically, E46K aSyn reduced JNK1 phosphorylation, which led to a downstream cascade effect of reduced Bcl-2 phosphorylation and increased association of Bcl-2 with Beclin 1 (Yan et al., 2014). As Beclin 1 remains bound to Bcl-1, it does not complex with hVps34 to stimulate autophagy. Pharmacological inhibition of JNK1 led to similar effects seen with E46K, suggesting this is indeed JNK1-dependent autophagy and the mammalian target of rapamycin (mTOR) pathway is not involved (Yan et al., 2014). How E46K aSyn inhibits JNK1 phosphorylation is unclear. Direct binding to Beclin 1 may be possible, as WT aSyn is capable of interacting with Beclin 1 (Decressac et al., 2013). Interestingly, CMA activity (measured by GAPDH activity) is increased in E46K-expressing cells, perhaps as a compensatory effect (Yan et al., 2014).

### A30P

The half-life of A30P aSyn is comparable to WT aSyn both *in vitro* and *in vivo* (Li et al., 2004), but it has an impact on the ALP system that may make this missense mutation causative for early onset familial PD. Like A53T, the A30P mutant impairs CMA (Cuervo et al., 2004). A30P blocks CMA due to its high binding affinity to the lysosomal membrane that prevents degradation of itself and other substrates. In this way, A30P aSyn behaves like A53T aSyn and DA-aSyn (Cuervo et al., 2004), although an earlier study showed that A30P colocalized with lysosomes in PC12 cells (Webb et al., 2003). The effects of A30P on autophagy mirror that of the E46K mutant.

In primary VM neurons transfected with A30P aSyn AAV vectors, aggregated p62 increased in a manner similar to that of the E46K mutant aSyn (Yan et al., 2014; Lei et al., 2019). A30P did not alter p62 mRNA levels, and 3-MA treatment increased p62 in both the mutant and empty AAV vector cells, supporting the conclusion that the insoluble aggregates were due to A30P-mediated autophagy inhibition (Lei et al., 2019). Like E46K, A30P also reduced LC3-II synthesis (but not degradation), suggesting a similar mechanism of impaired autophagosome formation (Yan et al., 2014; Lei et al., 2019). However, Baf treatment increased LC3-II levels, indicating that A30P inhibits autophagosome fusion with the lysosome (Lei et al., 2019).

Mechanistically, A30P increases the transcription factor ZKSCAN3 associated with genes responsible for autophagosome formation. This increase is paralleled by inhibition of autophagy. A30P-expressing VM neurons treated with short hairpin RNA (shRNA) against ZKSCAN3 had restored levels of LC3-II and less p62 aggregation. This suggests that A30P expression inhibits autophagy via ZKSCAN3; this is independent of the mTOR pathway, as rapamycin was unable to antagonize the effects of mutant aSyn. However, the decrease in phosphorylated JNK1 is similar to the effect of the E46K mutant. The data suggest that inactivation of JNK1 by A30P increases the activity of ZKSCAN3, which then translocates to the nucleus (Lei et al., 2019). Yet, as with E46K, there are several missing links in this mechanistic pathway.

Taken together, the existing data indicate that aSyn missense mutations result in a toxic gain of function, that is partly reflected by the effects of mutant aSyn on ALP. Despite being the first identified familial PD mutation, evidence for the signaling mechanisms of A53T toxicity toward the ALP is lacking. What is clear is that A53T inhibits CMA with some additional effects on autophagy in differentiated SH-SY5Y cells (Xilouri et al., 2009), which may include elevated lysosomal pH (Cuervo et al., 2004).

The mechanistic evidence for autophagy impairment is most complete for A30P and E46K. Autophagy inhibition by these mutants is mTOR independent and instead relies on inhibition of JNK1 phosphorylation upstream of autophagosome formation. The A30P mutant appears to prevent autophagosome fusion with the lysosome, whereas the E46K mutant prevents autophagosome formation. Both A30P and E46K localize inside lysosome as observed with fluorescence microscopy; however, evidence for CMA inhibition by A30P argues against this. CMA inhibition *in vitro* suggests that A30P, like A53T, binds with high affinity to the lysosomal membrane but does not translocate or allow other proteins to enter the lysosome. While there is some evidence for CMA alteration in E46K mutants, we will have to wait for clarifying studies. However, care must be taken in designing future studies and interpreting the findings, as different cellular background and differentiation states can yield variable results (Xilouri et al., 2009).

## aSyn Proteoforms: From Partial Proteolytic Degradation to Effects on Lysosomal Function

The lysosome has a role in removing aSyn from the cytoplasm to maintain the homeostatic balance between production and clearance. There is compelling evidence that aSyn proteoforms modulate detrimental effects on the ALP system, precipitating dysfunction and ultimately disease. However, incomplete lysosomal proteolysis of aSyn may be responsible for generating disease-associated proteoforms that could further impair the lysosome, leading to broader perturbations at the cellular level. We know less about how the lysosome can influence the emergence of non-canonical aSyn proteoforms. In this section, we review the existing data on lysosomal-induced aSyn proteoforms, discuss the questions and implications, and propose how this nascent area of research could move forward.

The lysosomal enzyme CtsD—and risk allele for PD (Robak et al., 2017) —degrades aSyn, but only at the hydrophilic C-terminus (Sevlever et al., 2008). CtsD removal of this section increases aSyn’s net hydrophobicity and aggregation potential (Liu et al., 2005; Ulusoy et al., 2010). In an acidic environment such as the lysosome, the C-terminally truncated aSyn proteoform is susceptible to amyloid formation (Uversky et al., 2001; McGlinchey and Lee, 2015). Pharmacological treatments that increase lysosomal pH from its more acidic basal state suppress aSyn aggregate formation (Tsujimura et al., 2015).

Evidence from neural crest cell-derived dopaminergic neurons suggests that glucocerebrosidase (GCase) is required for normal CtsD function (Heinrich et al., 1999; Yang et al., 2020). GCase mutations are the most common risk factor for PD, and the mutations studied by Yang and colleagues reduced both CtsD protein levels and enzymatic activity. The consequence of functionally impaired CtsD was increased monomeric aSyn levels (Crabtree et al., 2014; Yang et al., 2020). Yang and colleagues found no evidence of higher-order aggregates despite a ∼48% increase in aSyn relative to controls. However, oligomer levels increased in undifferentiated SH-SY5Y lines expressing inactive mutant CtsD (Crabtree et al., 2014) and CtsD knock-out mouse brain (Cullen et al., 2009). Similar results were found in primary cortical neurons and H4 neuroblastoma cells with shRNA-mediated knockdown of GCase, as well as in hiPSC-derived dopaminergic cells from mutation carriers (Mazzulli et al., 2011). The only exception to this was a study using 3D5 human B-cells under iron-induced oxidative stress that reported increases in aggregates containing a C-terminally truncated aSyn proteoform and a concomitant increase in CtsD activity (Takahashi et al., 2007).

**FIGURE 2:**
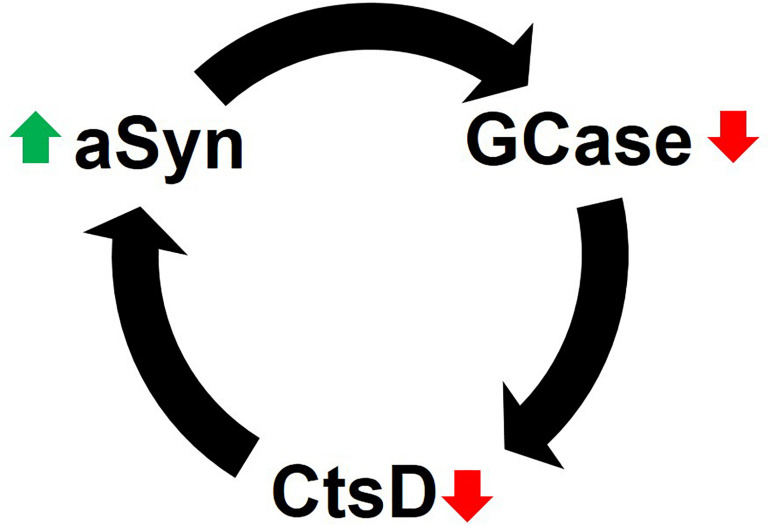
Reciprocal feedback loop of GCase inhibition and aSyn accumulation. CtsD functionality depends on GCase activity. When GCase activity is diminished, the lysosomal enzyme CtsD, which plays a role in aSyn degradation becomes limited. This leads to the accumulation of aSyn in cells. Likewise, elevated levels of aSyn (via multiple mechanisms) inhibits GCase to either initiate or enhance the destructive positive feedback loop of lysosomal impairment and aSyn accumulation (see text for details).

Together the data suggest that impaired GCase limits CtsD functionality, which in turn promotes aSyn accumulation and aggregation due to insufficient proteolysis (Figure 2). Interestingly, high levels of aSyn (i.e., 10 mM) inhibit GCase *in vitro* through physical interactions in lipid vesicles (Yap et al., 2013) and under lysosomal conditions (i.e., pH 5) (Yap et al., 2011). GCase inhibition would ultimately lead to inhibition of CtsD and elevated aSyn levels in a reciprocal feedback loop (in line with evidence by Yang et al., 2020) or reduced aggregated and truncated aSyn (based on data from Takahashi et al., 2007). However, supporting the work of Yang et al. (2020), PD brain and cerebrospinal fluid (CSF) have decreased CtsD levels irrespective of GCase mutation status (Parnetti et al., 2017; Moors et al., 2019). Mazzulli and colleagues reported that increased levels of aSyn depleted GCase, and GCase functional deficiences could also increase aSyn in a vicious, self-propogating feedback loop (Mazzulli et al., 2011).

It is important to emphasize that nearly all these studies were conducted in aSyn overexpressing cell lines. In non-over expressing lines, heterozygous CtsD was sufficient to degrade aSyn (Bae et al., 2015). However, if endogenous levels of aSyn are high, a partial CtsD deficiency increases aSyn aggregation via reduced lysosomal degradation (Bae et al., 2015). Yet, if the inhibition of CtsD—through GCase or CtsD mutations—promotes aSyn accumulation, how does CtsD that can only partially degrade aSyn produce an aggregation-prone C-terminally truncated species (Sevlever et al., 2008; McGlinchey and Lee, 2015) not adversely affect the lysosome or cell?

The answer appears to be through the concerted actions of other lysosomal enzymes (Figure 1). Cathepsin B (CtsB), L (CtsL), and K (CtsK) degrade aSyn, inhibiting its aggregation *in vitro* (McGlinchey and Lee, 2015; McGlinchey et al., 2020). Using recombinant aSyn monomers and fibrils, McGlinchey and colleagues mapped the CtsL cleavage sites. Compared to monomeric aSyn, fibrillar aSyn took much longer to degrade, and this only occurred after the enzyme removed significant portions of the hydrophilic C-terminus (McGlinchey et al., 2017). CtsK was superior at degrading recombinant aSyn PFFs compared to CtsL and removed both the N- and C-terminal portions of aSyn before full degradation could occur (McGlinchey et al., 2020). Using aggregation-prone proteoforms aSyn1-122 found in LBs (McGlinchey et al., 2019) and A53T, which is partially resistant to cathepsins (McGlinchey et al., 2020).

In this context, CtsD may even initiate degradation limited to the C-terminus (Sevlever et al., 2008), particularly in the presence of lipids (McGlinchey and Lee, 2015), leaving CtsL to complete the degradation process or CtsK may act alone *in vivo*, both possibilities would need to be tested in future investigations. By comparison, monomeric aSyn had an even distribution of CtsL cleavage across its primary sequence after just minutes. Remaining monomer and fibril aSyn peptides lose their amyloidogenic capacity after CtsL or CtsK digestion (McGlinchey et al., 2017, 2020). Still, rat liver lysosome extracts only had a limited capacity to degrade aSyn fibrils, even though CtsL concentrations were comparable between *ex vivo* and *in vitro* experiments (McGlinchey and Lee, 2015).

CtsB is a risk allele for PD (Chang et al., 2017) that degrades aSyn fibrils *in vitro*, but only to a modest extent (McGlinchey and Lee, 2015). Inhibition of CtsB in aSyn-expressing HEK293 cells significantly blocked aggregate formation (Tsujimura et al., 2015). In this model, proteinase K (PK) was able to degrade *in vitro*-prepared aSyn fibrils, but *in vitro* CtsB digestion enhanced aggregation (Tsujimura et al., 2015). While the PK susceptibility of these fibrils suggests they do not mimic the PK-resistant versions found in the PD brain, it is nonetheless intriguing that CtsB enhanced aSyn aggregation in the lysosome in this cell-based assay (Tsujimura et al., 2015). The species generated from this were presumably C-terminally truncated, as western blotting with the C20 antibody (C-terminal epitope) was unable to recognize the protein represented on a Coomassie-stained gel (Tsujimura et al., 2015). These data seem to suggest that the moderate fibril-degrading activity measured by McGlinchey and Lee (2015) is responsible for generating aggregate-prone C-terminally truncated aSyn if other lysosomal enzymes do not assist in lysosomal degradation (McGlinchey and Lee, 2015; Tsujimura et al., 2015). In support of this, evidence from a *Caenorhabditis elegans* model demonstrated that CtsB overexpression was not protective (Qiao et al., 2008).

Studying C-terminally truncated aSyn in an A53T transgenic model of PD, McGlinchey et al. assessed whether it was present in the lysosomal contents of symptomatic and age-matched asymptomatic mice (McGlinchey et al., 2019). Symptomatic transgenic mice (∼16 months) had increased levels of full-length, pS129, and C-terminally truncated aSyn. The authors ascribed this to an overburdened lysosome unable to degrade excess and aggregated (due to the pS129 finding) aSyn since lysosomal enzyme activity was unchanged (McGlinchey et al., 2019). To confirm that the lysosome incompletely degraded fibrillar aSyn to produce C-terminally truncated species, the authors treated N27 cells with recombinant aSyn fibrils. The lysosomes extracted from cells exogenously treated with aSyn fibrils contained full-length aSyn and two proteoforms of C-terminally truncated aSyn at ∼12 and 8 kDa (McGlinchey et al., 2019). Most compellingly, full-length aSyn was diminished when N27 lysosomes were sampled 20 h later, but the 12 kDa band and other higher-order aggregates remained. These proteoforms aggregated much more rapidly than full-length aSyn and had more potent seeding capacity (McGlinchey et al., 2019). Supporting the role of C-terminally truncated aSyn, the MI2 mice containing the transgene of human truncated 1–120 aSyn and knockout of mouse aSyn showed aggregation and progressive dopaminergic deficit (Wegrzynowicz et al., 2019).

Lasmézas and colleagues observed a ∼12 kDa aSyn species in primary neurons, mouse brains, and PD patient brains (Grassi et al., 2018). The authors referred to this less abundant species as “pα-syn STAR” (α-synuclein truncated adamant and reactive). The lower molecular weight of pα-syn^∗^ was due to a 10-amino acid truncation at the C-terminus and a 15-amino acid loss at the N-terminus based on antibody epitope recognition. They found that full-length fibrils localized to the autophagosome, and pα-syn^∗^ was present inside LAMP1-positive lysosomes (Grassi et al., 2018). The authors propose that this novel proteoform arose due to incomplete proteolytic degradation within the lysosome. Furthermore, lysosomes that contain pα-syn^∗^ lost their acidic internal environment. Treatment with rapamycin enhanced pα-syn^∗^ levels, which were decreased with chloroquine (Grassi et al., 2018). Since rapamycin is an enhancer (and chloroquine an inhibitor) of autophagy, this confirms that pα-syn^∗^ is a product of incomplete degradation by lysosomal enzymes that are only enzymatically active at low pH. Since lysosomes containing pα-syn^∗^ lost their acidic pH, the sequence of events is clear: (1) the cellular autophagy machinery transports full-length aSyn to the lysosome; (2) lysosomal enzymes incompletely degrade aSyn, producing pα-syn^∗^; (3) and the presence of pα-syn^∗^ causes lysosomal dysfunction as evidenced by loss of the lysosomal pH gradient. Ultimately, this proteoform leaves the lysosome and enters the cytoplasm, where it is mitotoxic (Grassi et al., 2018). These findings agree with those of McGlinchey et al. (2019), as the authors treated neurons and mice with high levels of exogenous PFFs, but lysosomal enzyme activity was not measured. Another unexplored possibility is that even if enzyme activity remains the same, in the presence of excess aSyn, the lysosomal enzymes have lost harmonization with one another. The work of Martinez-Vicente and colleagues suggests this may be the case, as the matrix components of isolated rat lysosomes could degrade aSyn *in vitro* given sufficient time (Martinez-Vicente et al., 2008).

Overall, the evidence suggests interplay among lysosomal enzyme activities. Inconsistencies or breakdowns in this interplay appear to result in increased aSyn levels. Based on molecular weights, the canonical monomeric proteoform is present, as well as aggregated proteoforms. Most notable is the consistent presence of C-terminally truncated forms and a novel ∼12 kDa proteoform detected by multiple groups (Takahashi et al., 2007; Grassi et al., 2018; McGlinchey et al., 2019). However, it seems in all these studies that elevated aSyn is a prerequisite for this effect given the use of overexpressing cell and animal models. The use of overexpression models is important for two reasons: it elicits effects that enable *in vitro* and *in vivo* study but also mimics the high cellular concentration of aSyn found in the PD brain. However, excessive a Syn accumulation represents a later and often symptomatic stage of PD. This confounds the origin of non-canonical aSyn proteoforms. Is excess aSyn truly a prerequisite to overwhelm lysosomes resulting in reduced proteolytic efficiency and toxic aSyn? Are lysosomes in the aging brain no longer efficient? If so, does this inefficiency yield toxic proteoforms that further damage the lysosome in a reciprocal feedback loop?

Investigations into how the lysosome may be a production center for toxic non-canonical aSyn proteoforms have only begun to emerge in the last 5 years. More work is need to: (1) identify other additional species using methods such as top-down mass spectrometry (Patrie, 2016; Wildburger et al., 2017; Smith and Kelleher, 2018; Schaffer et al., 2019); (2) understand mechanisms of formation; and (3) determine how aSyn escapes the lysosome. Importantly, these studies need to be done comparing physiological vs. increased aSyn levels to address the critical question of whether excess aSyn is necessary to prompt lysosome-generated non-canonical aSyn. Answering these questions will allow us to move forward and examine commonalities shared with other synucleinopathies.

## Therapeutic Strategies Targeting Lysosomal Functions

As previously mentioned, there is no effective treatment to stop or halt PD progression and neurodegeneration. Therefore, developing novel therapeutics aimed at disease modification is a great medical need and the main research focus in PD (Richter, 2019). The above-described interplay between aSyn proteoforms and the lysosome suggests using a two-pronged approach for therapeutic strategies (Table 1). There are several approaches to combat aSyn pathology; reducing expression, inhibiting or reducing its aggregation, preventing its spreading, and enhancing its degradation are major aspects under investigation by several groups (Dehay et al., 2016; Wong and Krainc, 2017; Richter, 2019). Due to the wide spectrum of aSyn proteoforms, it is impractical to individually target each one. Deciphering those that are most relevant to PD is a more viable alternative strategy, but would require: (1) unbiased methods to screen all potential aSyn proteoforms, (2) methodical evaluation of proteoform toxicity *in vitro* and *in vivo* (assays of disease-relevant activity) (Hong et al., 2018), and (3) targeted drug development toward the implicated proteoform(s). Methods such as top-down mass spectrometry have greatly matured over the last 10 years and may well be sufficient for the challenge of unbiased screening of all aSyn proteoforms (i.e., proteins < 30 kDa). Even so, time is a limited factor of this strategy. Identifying all or nearly all species most relevant to PD is a monumental task that will not deliver novel therapeutics in a reasonable timeframe—though with biomarkers it could. We therefore hypothesize that future therapies toward PD and other neurodegenerative diseases will be at least in part molecular. A feasible means to reduce or eliminate aSyn and its diverse proteoforms is through RNAi-mediated strategies (Helmschrodt et al., 2017). Such an approach should reduce aSyn concentrations, thus limiting the potential for aggregation and minimizing PTMs that confer or enhance toxicity. One recent line of evidence in support of this approach comes from a study by Izco et al. (2019), showing that anti-aSyn short hairpin RNA delivered in RVG-exosomes reduced pathology in a PFF-induced mouse model for up to 6 weeks. Initial behavioral evaluations showed reduced motor deficits in treated mice, but other unanticipated outcomes cannot be excluded. We can speculate, that due to the high level of homology of aSyn with bSyn and even gSyn (see section “aSyn: Small Protein, Enigmatic Function, and Many Diseases” above), the impact of RNAi on a biological system might not be harmful, but only well-designed pre-clinical and clinical tests could confirm this.

**TABLE 1:** Examples of preclinical therapeutic strategies targeting lysosomal function.

Strategy	Target	Agent	Application route	Main effect	PD model	References
Chaperone	GCase	Isofagomine	Oral	Improved motor and non-motor function, less neuroinflammation, increased aSyn clearance, fewer olfactory deficits	SNCA transgenic mice (Thy1-aSyn, line 61)	Richter et al., 2014
Chaperone	GCase	Ambroxol	Oral	Increased GCase activity, reduction of aSyn levels	SNCA transgenic mice with absence of mouse snca	Migdalska-Richards et al., 2016
Chaperone	GCase	Ambroxol	Oral	Improved motor function, dopaminergic system recovery, reduction of aSyn pathology	6-OHDA rat model	Mishra et al., 2018
Chaperone	GCase	Ambroxol	Oral	Increased GCase activity	Healthy non-human primates	Migdalska-Richards et al., 2017
Small molecule	GCS	GZ667161	Oral	Reduction of glucosylceramide levels, amelioration of memory deficit, reduction of hippocampal aSyn aggregates	Gba^*D40*9V/D409V^ and A53T–SNCA mice	Sardi et al., 2017
Viral vector	TFEB	AAV-TFEB	Intracerebral	Prevention of behavioral impairment, protection of nigral DA neurons	AAV-aSyn rat model	Decressac et al., 2013
Viral vector	Beclin 1	AAV-Beclin 1	Intracerebral	Prevention of behavioral impairment, protection of nigral DA neurons	AAV-aSyn rat model	Decressac et al., 2013
Viral vector	LAMP-2A	AAV-LAMP-2A	Intracerebral	Amelioration of dopaminergic neurodegeneration, reduction in total aSyn levels	AAV-aSyn rat model	Xilouri et al., 2013
Nanoparticles	Lysosome	Acidic nanoparticles	Intracerebral	Restored lysosomal pH and lysosomal function (all models), inhibited dopaminergic cell death (MPTP-treated mice)	MPP+-treated cells, ATP13A2 mutant fibroblasts, and MPTP-treated mice	Bourdenx et al., 2016
Nanoemulsions	Lysosome	Acidic nanoemulsions	Intracerebral, retro-orbital injections	Restored lysosomal pH and lysosomal function (*in vitro*), biodistribution into the SNc and VTA (WT mice)	ATP13A2- mutant M17 cells, and WT mice	Prevot et al., 2018

*AAV, adeno-associated virus; aSyn, alpha synuclein; DA, dopamine; GCase, β-glucosidase; GCS, glucosylceramide synthase; LAMP, lysosomal-associated membrane protein; MPP+, 1-methyl-4-phenylpyridinium; MPTP, 1-methyl-4-phenyl-1,2,3,6-tetrahydropyridine; 6-OHDA, 6-hydroxydopamine; TFEB, transcription factor EB; VTA, ventral tegmental area.*

A second and possibly concurrent therapeutic approach would employ strategies that enhance lysosomal function to counteract aSyn (Spencer et al., 2009; Mazzulli et al., 2011, 2016a; Xilouri et al., 2016). This would require a firm understanding of which ALP mechanisms are altered by aSyn. However, as there is now evidence that the lysosome may in fact be partly responsible for generating toxic aSyn proteoforms, such an approach must be carefully titrated and validated due to the risk of exacerbating disease pathogenesis. Below we describe examples of therapeutic strategies targeting lysosomal function (Table 1).

One recently proposed strategy is the use of small-molecule chaperones such as isofagomine and ambroxol. These are orally available and specifically target the misfolded GCase, thus increasing its trafficking to lysosomes (Sawkar et al., 2006; Valenzano et al., 2011). For example, isofagomine can increase GCase activity both *in vitro* and *in vivo* (Sun et al., 2012; Richter et al., 2014; Sanchez-Martinez et al., 2016). Chronic oral administration of isofagomine to mice overexpressing human wild-type aSyn improved motor and non-motor function, abolished microglial inflammatory response in the SNpc, decreased aSyn expression, and reduced the number of small aSyn aggregates (Richter et al., 2014).

Marketed as an expectorant since the 1970s, ambroxol first demonstrated its beneficial effects by increasing GCase activity and restoring lysosomal function in GBA1 mutant fibroblasts (McNeill et al., 2014; Ambrosi et al., 2015). In the 6-hydroxydopamine rat model, chronic oral ambroxol administration improved motor functions, recovered the dopaminergic system, and reduced aSyn pathology (Mishra et al., 2018). After showing that daily oral administration reduced aSyn levels in transgenic mice and increased GCase activity in healthy non-human primates (Migdalska-Richards et al., 2016, 2017), thereby confirming that it crosses the blood-brain barrier (BBB) in different species, the way was paved for clinical trials. The initial results suggested that ambroxol was safe and well tolerated in 18 patients with moderate PD. Ambroxol showed CSF penetration and target engagement, and aSyn levels were increased in CSF (Mullin et al., 2020). There is an ongoing placebo-controlled clinical trial testing the effect of ambroxol on cognitive and motor symptoms in 75 PD dementia patients (ClinicalTrials.gov identifier: NCT02914366) (Silveira et al., 2019).

Chaperones potentially inhibit enzyme function and therefore require specifically titrated dosing regimens. NCGC00188758, a small molecule which directly activates GCase, was recently demonstrated to partially reverse aSyn-induced cellular pathology and neurotoxicity in hiPSCs from PD patients (Mazzulli et al., 2016b).

Beyond targeting GCase itself, therapeutic inhibition of the enzyme glucosylceramide synthase (GCS) that catalyzes the synthesis of GCase’s substrate glucosylceramide (GlcCer) is a promising approach to increase lysosomal activity. There are currently two FDA-approved GCS inhibitors for treating GD (miglustat and eliglustat), but they have no effect on central nervous system pathology due to poor entry into the brain. Therefore, a novel orally available inhibitor of GCS named GZ667161 has been tested in preclinical models. GZ667161 was able to cross the BBB and reduced the substrate GlcCler as well as glucosylsphingosine in a mouse model of type 2 GD (Cabrera-Salazar et al., 2012). Moreover, chronic oral administration of GZ667161 in two mouse models of synucleinopathy reduced GlcCer levels, ameliorated memory deficits, and reduced hippocampal aSyn aggregates (Sardi et al., 2017). Venglustat, another brain-penetrant allosteric inhibitor of GCS, is currently being tested in a global Phase 2 trial to evaluate safety and efficacy in PD patients who are heterozygous for a GBA1 mutation (ClinicalTrials.gov identifier: NCT02906020), supporting this promising strategy of targeting GCS.

As detailed above, ALP dysfunction contributes to PD pathogenesis. Therefore, viral vector-mediated overexpression of ALP regulators such as the transcription factor EB (TFEB) and Beclin 1 is another strategy to develop disease-modifying therapies for PD. Overexpression of TFEB or Beclin 1 prevented behavioral impairment and protected nigral DA neurons in rats with AAV vector-mediated overexpression of human wild-type aSyn, a well-established PD model (Decressac et al., 2013). Similar positive results including amelioration of dopaminergic neurodegeneration and lower total aSyn levels were observed when overexpressing LAMP-2A via a viral vector in the same animal model (Xilouri et al., 2013). In an MPTP-induced mouse model of PD, TFEB-AAV vector delivered stereotaxically to the right SNpc increased autophagy markers LAMP-1, CtsD, and LC3-II/LC3-I ratio ∼57–133% and activated protein synthesis and pro-survival pathways. Concurrently, SNpc volume, area, and intracellular TH increased, but whether this also reflected improved motor function is unknown (Torra et al., 2018). Similar results were achieved in rats with AAV vector-delivered overexpression of human A53T aSyn to the SNpc (Arotcarena et al., 2019). In this study, treatment was able to prevent behavioral impairments in the ipsilateral rotations and left paw use examinations. While the study highlights the importance of neuronal-specific targeting of TFEB more behavioral analysis is needed. It is also unclear if AAV-TFEB is able to prevent aSyn pathology and motor deficits *after* aSyn accumulation since both AAV-A53Tα-syn/AAV-TFEB were co-administered 1:1 unilaterally (Arotcarena et al., 2019).

Besides the aforementioned approaches, directly targeting lysosomal activity appears to be an encouraging disease-modifying strategy. FDA-approved acidic nanoparticles (aNPs) have been reported to traffic to lysosomes and affect lysosomal pH (Baltazar et al., 2012). Bourdenx et al. (2016) tested the effect of aNPs in MPP+ (1-methyl-4-phenylpyridinium)-treated cells, ATP13A2 mutant fibroblasts, and MPTP (1-methyl-4-phenyl-1,2,3,6-tetrahydropyridine)-treated mice. aNPs restored lysosomal pH and function in all three models. Additionally, aNPs inhibited dopaminergic cell death in MPTP-treated mice. In a more recent development, Poly(DL-lactide-co-glycolide) (PLGA) nanoemulsions were able to restore lysosomal pH with better brain distribution compared to previous iterations (i.e., acidic nanoparticles) (Prevot et al., 2018). Of course, therapeutic approaches to PD are not limited to aSyn RNAi and lysosomal enhancement (see also other contributions to this special issue). What is clear is that a multitargeted approach with early intervention is required.

## Conclusion and Future Directions

**FIGURE 3:**
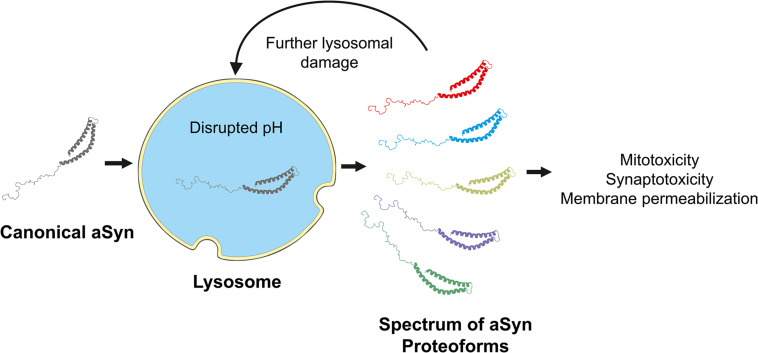
Impair lysosomal degradation produces a spectrum of aSyn proteoforms. “Canonical” full-length aSyn is degraded via the autophagy lysosomal pathway. However, disruptions in lysosomal pH, enzyme activity or harmonization, lead to the incomplete digestion of the canonical aSyn proteoform. The resulting novel or non-canonical proteoforms, typically truncated one or both termini, may either accumulate in the lysosome further impairing functionality or escape into the cellular milieu.

Collectively, these results indicate that there is a significant bidirectional relationship between the lysosome and aSyn (Figure 3), which appear locked in a maladaptive feedback loop. Existing proteoforms (non-ALP generated) directly damage the lysosome, but the lysosome itself is responsible for producing toxic aSyn proteoforms that remain internalized in the organelle or are released into the cellular milieu (Dehay et al., 2012; Bourdenx et al., 2014; Grassi et al., 2018). The latter half of this pathological loop has emerged within the last 5 years thanks to several exciting studies (Cullen et al., 2009; Mazzulli et al., 2011; Crabtree et al., 2014; Grassi et al., 2018; McGlinchey et al., 2019; Yang et al., 2020), but more remains to be elucidated in this nascent research area. One of the most remarkable emerging features is the surprising diversity in aSyn proteoforms, which is likely still underappreciated as other PTMs may be involved in lysosomal dysfunction. While the catalog of aSyn proteoforms is large and continually increasing, characterizing their impacts on disease progression is critical for developing rational and urgently needed treatment targets. Therapeutic development must go hand-in-hand with the identification of suitable biomarkers for clinical trial screening, diagnosis, prognosis, and response to interventions (i.e., companion diagnostic tests). Unbiased discovery of aSyn proteoforms will reveal which are associated with pathological and molecular changes that correlate with PD progression and severity. These proteoforms—especially if they are unique to PD and not seen in other synucleinopathies—would serve as templates for novel, specific positron emission tomography ligands for diagnostic imaging. In summary, a closer look at aSyn proteoforms and lysosome dysfunction in PD has revealed a complex, unsteady pathogenic loop that could very well be driving this multifaceted and progressive neurodegenerative disease.

## Author Contributions

NW wrote the first draft with text, table, and figure contributions by A-SH and AS. FR extended and revised the draft. NW and FR generated the final manuscript. All authors contributed to the article and approved the submitted version.

## Conflict of Interest

The authors declare that the research was conducted in the absence of any commercial or financial relationships that could be construed as a potential conflict of interest.
